# Items, Selections and Remarks

**Published:** 1870-03

**Authors:** W. W. Miner


### Items, Selections and Remarks.

BY W. W. MINER, A. B.

The treatment of Syphilis by the hypodermic injection of mercurial solutions has recently been brought to notice, though it was used six years since. *Dr. Good*, in writing from Paris, says that the injection of Calomel invariably produces abscesses and induration of the parts injected ; corrosive sublimate is very much less likely to produce these results. M. Liegeois employs the following solution: Corrosive sublimate, twenty centigrammes; hydro-chlorate of morphine, ten centigrammes; distilled water, one hundred grammes. Mix. Twenty drops of this solution is injected into the cellular tissue twice a day for thirty-five days, and this constitutes the whole treatment. The advantage of this method is that the action of the mercury is rapid and reliable. Pain is, however, caused in the injected parts, and gastritis often occasioned, but in cases where patients, willing to bear the sting, are anxious to see their secondary symptoms rapidly disappear, he would resort to the hypodermic method.——Prof. Boeck of Christiana, Norway, an eminent syphilologist, has taken residence in New York City, where he is continuing his experiments in syphilization. An article by him, published in the *American Journal of Syphilography and Dermatology* is thus summed up by the editor of the *Gazette*. "Dr. Boeck states :—1. That he only uses syphilization as a curative means and not as a prophylactic, as originally proposed by Auzeas Turenne. 2. That he uses, by preference, the matter from indurated chancre, which must previously be irritated, to obtain efficient inoculations. 3. He does not object to the use of matter from a soft chancre, as experience has taught him that the result is the same. 4. From his diagnostic and curative inoculations, he is convinced of the unicity of the virus of the two forms of primary sore. 5. That during the course of the inoculations, the syphilitic symptoms gradually subside, and as a rule have entirely disappeared when the virus fails to produce appreciable results.

Dr. Stimpson, at a recent meeting of the Chicago Academy of Sciences, reported the results of three months experiments on the preservative qualities of Carbolic Acid as compared with that of Alcohol. He found that a solution containing two and one-half per cent of carbolic acid, gave a fluid that equalled alcohol in the preservative power, and at less than one-twentieth its cost. While such a solution leaves the specimen intact and perfect, a solution of five per cent strength, (which is almost a saturated one) will soon destroy it. The specimen should first be placed in a solution of one-half per cent strength, which may be daily changed for a stronger one.——The vulcanized rubber plate in which artificial teeth are now commonly set, is said to be injurious to health on account of the vermilion or mercuric trisulphide which is used to give the flesh color to it.—— There are eight institutions in the United States for the training of idiots. The largest is at Syracuse, N. Y., and it has 150 pupils.—*Pacific Medical Journal.*

Dr. Von Graefe has returned to Berlin from his tour in search of health, in Italy, greatly benefitted, and as enthusiastic as ever. —— M. Ricord has been officially appointed consulting physician to the Emperor. —— A movement is being made for the placing of a bronze statue of Harvey, in Central Park, New York City.—— Dr. Erasmus Wilson has received the appointment of Professor of Dermatology in the Royal College of Surgeons, England. His Journal of Cutaneous Medicine was discontinued in January. —— Nelaton calls Napoleon's disease "vesical hemorrhoids."——One hundred and fifty babies have been left in the basket at the New York Foundling Asylum since the twentieth day of November last.

A method of concentrating sulphuric acid without the use of platinum stills has been devised by M. Costello. It consists in letting the acid drop through a heated tower among bits of silica. —— A galvanic battery, whose elements are zinc, peroxide of manganese and hydro-chlorate of ammonia, is said to be in extensive use in Europe. —— During the storing of potatoes in the cellar, up to January, the amount of starch is increased; towards spring it diminishes on account of its change into glucose and dextrine.—*Pharmacist.*——Washington University, Baltimore, graduated a class of forty-eight physicians in February.——Seventy medical degrees were conferred by the College of Physicians and Surgeons, at New York, last month.——The University of Louisville conferred the degree of M. D. upon ninety-two graduates, March 1st.

The Parisian authorities have prohibited the use of copper salts in pickles and aniline in confectionery.—Small-Pox is prevailing in Paris.—The Senate, notwithstanding the efforts of Mr. Sumner, by a small majority, voted not to repeal the charter of the Medical Society of the District of Columbia.—An effort is being made to establish a Presbyterian Hospital in Philadelphia.——The *Pall Mall Gazette* states that a young woman has died from the effects of chloroform, at Hospital at Alloa, the administrator having been Sir James Y. Simpson.——The first to make human dissections, was Vesalius, of Spain. He was sentenced to the Holy Land, and died at sea on his return homeward. To the illustrious Velpeau we owe the first attempts at making anatomy a science.—*Record.*——A materialistic surgeon of Paris recently exhibited to one of his friends, an instrument the handle of which was carved in bone. "Do you know," he asked, "of what this handle is made?" "Of ivory, I suppose." "No," said the doctor, while tears almost choked his voice, "it is the thigh-bone of my poor aunt."—*Reporter.*

